# The Risk of Mercury Exposure to the People Consuming Fish from Lake Phewa, Nepal

**DOI:** 10.3390/ijerph110706771

**Published:** 2014-06-27

**Authors:** Devna Singh Thapa, Chhatra Mani Sharma, Shichang Kang, Mika Sillanpää

**Affiliations:** 1 Human and Natural Resources Studies Centre, Kathmandu University, P.O. Box 6250, Kathmandu, Nepal; E-Mail: charas07@gmail.com; 2 Key Laboratory of Tibetan Environment Changes and Land Surface Processes, Institute of Tibetan Plateau Research, Chinese Academy of Sciences, Beijing 10085, China; E-Mail: shichang.kang@itpcas.ac.cn; 3 Laboratory of Green Chemistry, Lappeenranta University of Technology, Sammonkatu 12, FI-50130 Mikkeli, Finland; E-Mail: mika.sillanpaa@lut.fi; 4 State Key Laboratory of Cryospheric Sciences, Cold and Arid Regions Environmental and Engineering Research Institute, Chinese Academy of Sciences, Lanzhou 730000, China

**Keywords:** fish consumption, mercury exposure, Lake Phewa, Nepal

## Abstract

The risk of mercury exposure through consumption of fish from Lake Phewa, Nepal was investigated. A total of 170 people were surveyed to know their fish consumption levels. The weekly mercury (Hg) intake in the form of methylmercury (MeHg) through fish was calculated by using the data on average MeHg concentrations in fish, the average consumption of fish per week, and an average body weight of the people. Hotel owners were consuming significantly high amounts of fish, followed by fishermen, in comparison to the government staff, army/police, locals and others (visitors). Some individuals exceeded the Provisional Tolerable Weekly Intake (PTWI) of 1.6 µg per kg body weight of MeHg (FAO/WHO). The minimum intake of MeHg (0.05 µg/kg/week) was found in the visitors (others) category, whereas the hotel owners had the maximum intake (3.71 µg/kg/week). In general, it was found that a person of 60 kg can consume at least 2 kg of fish per week without exceeding PTWI such that it does not pose any health risk associated with Hg poisoning at the present contamination level. Hg based PTWI values for Nepal has not been proposed yet in fishery resources so as to reduce health risk of the people.

## 1. Introduction

Exposure to toxic metals such as mercury (Hg) is one of the universal health problems associated with the consumption of aquatic resources (mainly fish) since the onset of modern civilization [1,2,3]. There is a clear link between atmospheric Hg loadings due to anthropogenic activities and MeHg accumulation in fish tissues [4]. Due to the semi-volatile nature of Hg, it has a long-range transport capacity which results in its widespread atmospheric distribution, hence making it a global pollutant [5]. Cold condensation effects, as well as wind directions bring it back, a process also influenced by dry as well as wet deposition at high latitudes [5] (e.g., Polar Regions) and high altitudes [6] (e.g., the Tibetan Plateau and the Himalayas), passing ultimately onto the aquatic resources [7]. The aquatic medium is one of the best places where Hg is converted by sulfate-reducing bacteria [8] into an organic form called methylmercury (MeHg), a highly neurotoxic compound [9]. In addition, MeHg has bioaccumulation capacity in the biota, as well as biomagnification capacity across the food chain [3,10,11,12,13] and its concentration increases many fold in top predators compared to the ambient waters. Therefore, top predator fishes generally have very high concentrations of MeHg in their bodies [13]. As MeHg transfers from one biota to another through food, fish is the main route of Hg exposure to human beings [13,14]; and studies of this topic have covered large areas of the globe.

Many researchers have indicated that the main form of Hg found in the biota is methylmercury (MeHg), a highly neurotoxic compound [9] that causes damage to the central nervous system. Since the main susceptible groups for Hg poisoning are growing fetus and growing children (*i.e*., undergoing brain development), women of reproductive age and small children are regarded as the most susceptible targets to Hg poisoning [15,16,17,18,19]. It has been proven that Hg is bound to proteins such as muscle, which is the main edible part of fishes, and its levels there cannot be reduced or removed by cooking [20]. Although the issue of Hg pollution has gained worldwide attention, such studies are still lacking in Himalayan regions of the world. Pokhara is one of the busiest cities in Nepal, with a huge influx of tourists, a group who readily participates in the consumption of fishes obtained locally (mainly from Lake Phewa and others like Begnas and Rupa). For that reason, the present study investigates the possible exposure of people to Hg pollution through the consumption of fish among those individuals who live near Lake Phewa and among those who consume fish products from the same lake.

## 2. Materials and Methods

**Figure 1:**
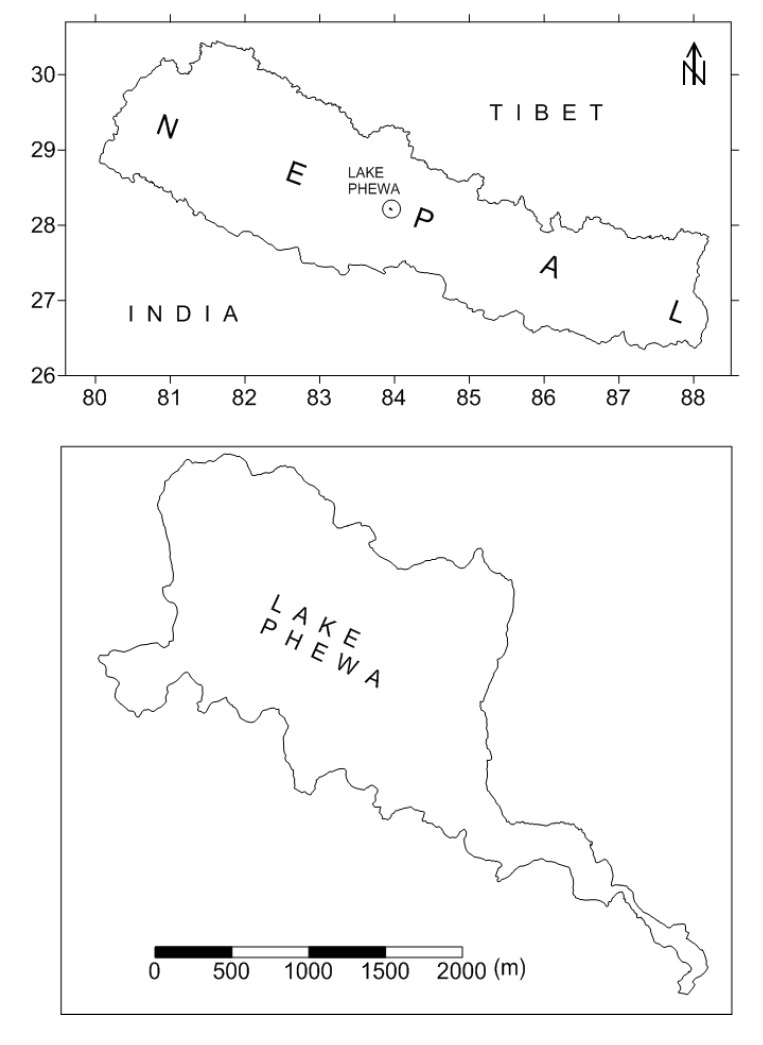
Location of Lake Phewa, the Study Area of the Present Investigation.

Lake Phewa is situated approximately at the centre of the country at an elevation of 782 m (Figure 1). It has a surface area of 4.35 km^2^ with an average depth of 8.6 m [21]. The maximum depth of the lake measured by Sharma *et al.* [22] was 22.5 m. There are around 28 species of fishes in the lake. Four fish species, viz., tilapia (*Oreochromis niloticus*) spiny eel (*Mastacembelus armatus*), African catfish (*Clarian gariepinus*) and sahar (*Tor putitora*) are regarded as commercially important fishes based on their sale in the local markets [22].

Three major fish species studied by Sharma *et al.* [22] were used to derive the levels of MeHg contamination in fish tissues to calculate the potential exposure levels to humans (the only available data on fish MeHg from Nepal yet; *n* = 58). These fish include: tilapia (*n* = 20), spiny eel (*n* = 19) and African catfish (*n* = 19). The important fish species sold in the local market during the present study period were the same ones, with tilapia representing the dominant species. Although the existence of tilapia in Lake Phewa was only noticed after 2,000 A.D., it has since dominated the total landings of the fisheries at the time of study.

A total of 170 individuals who consumed fish products from Lake Phewa were interviewed to know their eating habits. The purposive sampling technique was used as the samples needed to fulfill specific criteria for selection, e.g., the main criteria for inclusion of participants in the study were adult consumers of fish from Lake Phewa. Based on this criterion the population was divided into six categories: Staff (from the fisheries research center), Hotel owners, Fishermen, Locals, Army/Police, Others (visitors). The sample size was based on the saturation point principle, *i.e.*, interview to a certain group was stopped when no additional information could be obtained. The fish consumption habits of the participants were obtained by using food frequency questionnaires, mainly to get data on weekly fish intakes. As the respondents did not show any preference to the type of fish and informed that they consume whatever is available in the market (mainly tilapia), they were requested to provide an average weekly intake. A face-to-face questionnaire survey was conducted in local language so as to obtain data on fish diet consumption. The interviewer stayed in the study area for a month so as to administer the questionnaires. Before actual interview to the survey population, the questionnaires were pre-tested to know if the general public understood the questions properly. This has increased the validity of the questionnaires which enabled the researchers to get the information without being deviated.

The exposure level was determined by calculating Provisional Tolerable Weekly Intake (PTWI) established by Joint Expert Committee on Food Additives (JECFA, 1.6 µg/kg) and National Research Council (NCR: 0.7 µg/kg) of the U.S. [23]. The MeHg (µg/kg) intake per kg body weight per week was calculated by using the formula:

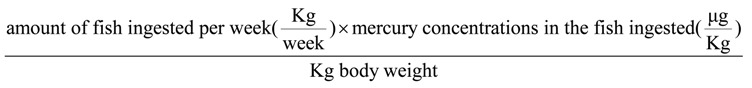



In this way, the intake of MeHg (µg/kg body weight) was compared to the PTWI established by JECFA and USNCR. Due to the skewed distribution of the data, a non-parametric Kruskal-Wallis test was performed to compare the consumption of fish by different categories of the people. Statistical tests were performed by SigmaStat and the plots were created using the SigmaPlot software. Statistical tests were considered significant at a *p* < 0.05 level, unless otherwise indicated.

## 3. Results and Discussion

The list of fish consumed by different categories of people during this study is provided in Table 1. Hotel owners consumed significantly higher amounts of fish compared to all other categories (Kruskal Wallis Test: H = 25.738; d.f. = 5; *p* < 0.001; Table 1). Similarly, the same test revealed that fishermen ranked second as they consumed significantly higher amounts of fish compared to staff.

One of the main reasons for the higher consumption of fish by the hotel owners in the present study could be due to the existence of left-overs that they felt compelled to eat instead of disposing of it or saving it for the next day. As in other studies, profession is not the only factor that influences the consumption of fish, but gender and village distribution also affect the consumption [24]. Culture and religion are the other factors that motivate and compel people for higher fish consumption irrespective of their knowledge on contamination [25].

**Figure 2:**
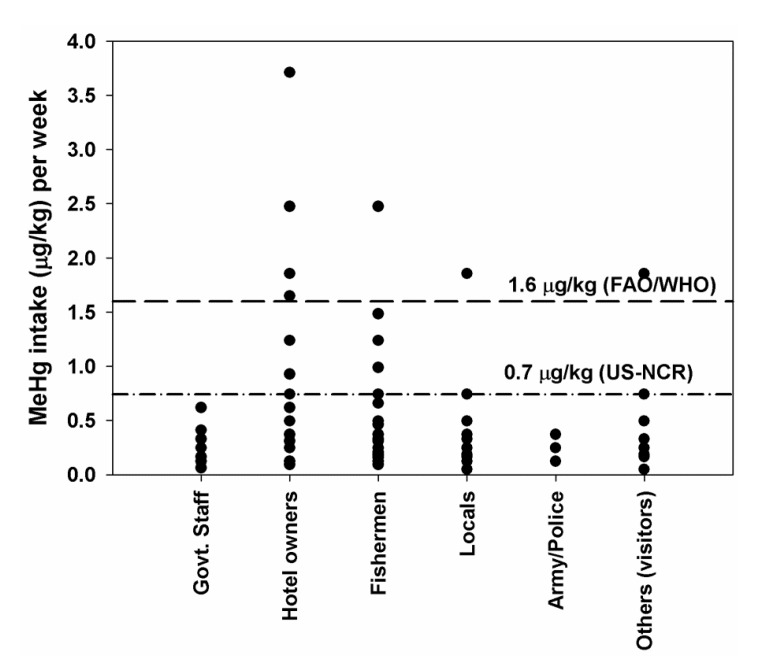
Risk of MeHg intake by individuals based on their fish consumption habits from Lake Phewa.

The individual fish consumption rate differed among the groups. When MeHg intake was calculated based on the individual fish consumption rates, it was seen that some individuals among hotel owners, fishermen, locals and others (visitors) exceeded the PTWI values set by the FAO/WHO (1.6 µg/kg body weight). In addition, many individuals exceeded the PTWI values set by the US-NRC (0.7 µg/kg body weight) except two categories; govt. staff and army/police (Figure 2).

**Table 1:** Fish consumption (kg/week) and MeHg intake (µg/kg/week) from Lake Phewa by different categories of people (*n* = 170).

Categories of People	Food Consumption (kg/week)				MeHg Intake (g/kg/week)			
	Median	Average (± SD)	Min.	Max.	Median	Average (± SD)	Min.	Max.
Staff (*n* =26)	0.500	0.478 (±0.298)	0.125	1.25	0.247	0.236 (±0.148)	0.062	0.618
Hotel Owners (*n* = 35)	0.750	1.335 (±1.486)	0.188	7.5	0.371	0.660 (±0.735)	0.093	3.710
Fishermen (*n* = 45)	0.667	0.929 (±0.863)	0.188	5.0	0.330	0.460 (±0.427)	0.093	2.473
Locals (*n* = 32)	0.500	0.663 (±0.63)	0.1	3.75	0.247	0.328 (±0.311)	0.049	1.855
Army/Police (*n* = 20)	0.500	0.512 (±0.172)	0.25	0.75	0.247	0.254 (±0.085)	0.124	0.371
Others (visitors) (*n* = 12)	0.584	0.915 (±0.978)	0.1	3.75	0.289	0.452 (±0.484)	0.049	1.855

**Figure 3:**
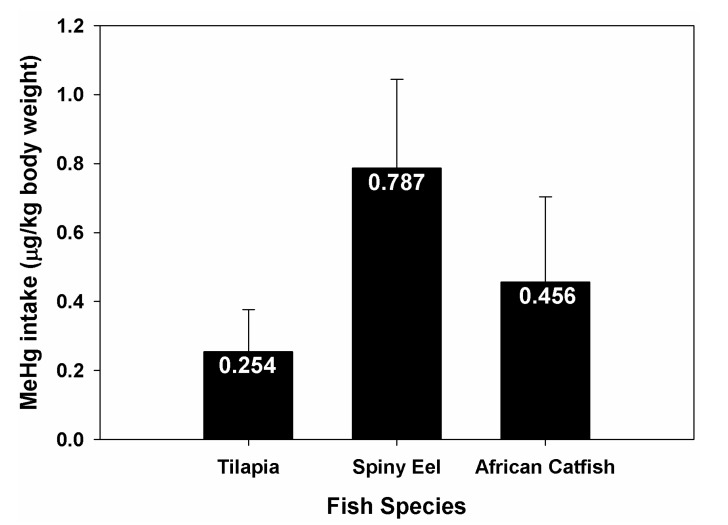
Intake of MeHg by consuming one kg of fish from Lake Phewa by a 60 kg person. The figures indicate average value and the error bars indicate standard deviation.

The investigation revealed that MeHg intake per kg body weight depends on the species of fish being consumed. For a person of 60 kg weight with a meal size of 250 gm of fish four times a week, MeHg intake is higher for spiny eel, followed by African catfish and tilapia the last (Figure 3). The lower MeHg content in tilapia was mainly due to its feeding behavior [26] as it consumes mainly plant materials which are lower in the trophic position [12,22,27]. Similarly, the higher MeHg intake resulting from consuming spiny eel is due to its predatory nature in which it occupies higher trophic position [22]. A person can consume 6.3 kg of tilapia, 3.5 kg of African catfish and 2 kg of spiny eel per week and still not exceed the PTWI established by the FAO/WHO (1.6 µg/kg body weight). However, the same person can consume only 2.7, 1.5 and 0.9 kg of the respective fishes per week to not exceed the 0.7 µg/kg body weight limit proposed by the US-NCR. The government should be responsible for obtaining information on the availability and contaminant levels for commonly eaten fish species so as to allow people to make informed decisions about the health risks from fish consumption [16,28]. Sometimes the people are compelled to choose chemical risk rather than giving up the consumption of contaminated fish due to their cultural and religious beliefs [25].

Awareness of people towards mercury poisoning by fish consumption differs from place to place [29]. Many nations have established their own PTWI levels as to make their people aware of the potential for Hg contamination in fish and to reduce health risks, whereas other nations follow the FAO/WHO recommendations [23]. This is true for most of the developed nations where PTWI levels vary with time as they generate more data [16,23,30]. The susceptible groups to Hg pollution such as pregnant-women and women of childbearing age were highly aware in the developed nations (e.g., US and the European nations) as the government issued advisories on contamination in food so as to protect the health of the general public [16,19,23]. A study in the U.S. indicated that the awareness among the susceptible group, particularly women, increased after the intervention of the government by providing consumption advice [31]. Such provisions sometimes may, however, lead to neglect the health benefits of eating a sufficient amount of fish [19]. Nepal has neither developed such a PTWI level nor has it officially followed the FAO/WHO guidelines. There is a need of monitoring levels of Hg contamination in fishery products from different parts of the country and establish a safe level for consumption nationwide.

## 4. Conclusions

Fish consumption from Lake Phewa varied significantly among different categories of people with highest consumption being by hotel owners, followed by fishermen. The risk of MeHg intake through fish consumption is thus higher for hotel owners compared to others. Tilapia is safer for consumption based on the Hg levels compared to other commercially important fishes from higher trophic positions. Nevertheless, the consumption of at least 2 kg of fish per week (among the three studied species) from Lake Phewa does not pose health risk due to Hg contamination based on the PTWI recommended by FAO/WHO. There is a need for investigation of fishery resources from different parts of the country so as to create a robust PTWI to protect people from the risk of Hg poisoning.
